# Laceration of the Cervix Uteri

**Published:** 1879-08

**Authors:** A. Reeves Jackson

**Affiliations:** Formerly Surgeon-in-Chief of the Woman’s Hospital of the State of Illinois; Fellow of the American Gynæcological Society, etc., etc.


CHICAGO MEDICAL

JOURNAL & EXAMINER.

VOL. XXXIX.—AUGUST, 1879.—No. 2.

[In these pages dimension and weight are expressed in terms of the Metric System, and temperature in degrees of the Centigrade Scale.]

### Original Communications.

### Article I.

LACERATION OF THE CERVIX UTERI. By A. REEVES JACKSON, A.M., M.D., etc., formerly Surgeon-in-Chief of the Woman's Hospital of the State of Illinois; Fellow of the American Gynæcological Society, etc., etc. Read before the Chicago Medical Society, July 7, 1879.

If Dr. Thomas Addis Emmet had done nothing more for the medical profession than tell its members what he has told them in reference to the subject of laceration of the cervix uteri, he would be entitled to their warmest gratitude. Not that he was the first to recognize the lesion as a distinct factor in the causation of uterine disease, for this had already been done, as early as 1856, by Dr. A. K. Gardner,* who speaks of "laceration of the os and cervix," as causes of ulceration, enlargement of the cervix, sterility, etc.: but to Dr. Emmet is due the credit of effectively calling attention to the frequency and pathological

*The Causes and Curative Treatment of Sterility, etc., by A. K. Gardner, A.M., M.D., New York, 1856.

importance of the injury, and, above all, of showing how to recognize and cure it. Indeed, he has written so much about it, and so identified himself with its history, that other observers have been able to do but little more than add to the number of his facts, corroborate his views and commend his methods.

Though it is not to the lot of all to be discoverers of important truths, all may aid in their dissemination; and I hold it to be the duty of those who have found themselves benefited and their hands strengthened by the observations and experience of others, to assist, so far as it may be in their power, in extending these benefits to their fellows. Herein lies the reason why, although I have no new facts to offer, nothing, indeed, but the meagre results of my own experience, I yet feel warranted in asking your attention to this subject; one whose interest and import are not, I am sure, duly appreciated by the profession at large.

If it were in my power to do so, I would like to make every member of this society, and every member of the medical profession, understand and realize that injuries to the uterine cervix during the process of parturition are not of rare and occasional happening, but that they are, on the contrary, of extremely frequent occurrence; that these lesions are not trivial in their nature and consequences, but that they are often of great and enduring pathological moment; that they are frequently the undiscovered cause of many of the derangements of health in women which have been and still are habitually attributed to other sources and conditions; that the venerable myth "ulceration," and its Fidus Achates, enlargement of the cervix, are so rare (except when the seat of malignant disease) that we may reach a ripe old age without seeing either of them; and that these terms, in such common use, may and should be in almost every instance rendered "laceration of the cervix." When these truths—for such I hold them to be—are known and accepted, there will not, I trust, be so many nervous, weak-backed, tired-out women as now crowd our waiting-rooms; we shall hear less of the uselessness of pessaries for "falling" wombs, less of amputation for enlarged ones; know less of failures to cure "the whites;" see less of pallid patients who wander—hopelessly at last—from one doctor to another, only to have repeated, by each one in turn, the "burning off" of the ulcers! This was the ordeal through which all womb-ailing women formerly passed, and through which thousands are passing now.

Until within the past two or three years the literature of cervical laceration was very scanty. In the autumn of 1862, Emmet* accidentally discovered the existence and importance of the injury, and devised an operation for its cure. Seven years later he published a paper,† in which the details of the operation were fully described. In November, 1874, he published another paper,‡ giving prominence to the frequency of the lesion, and the relation it bore to the etiology of pelvic disease; and in December, 1876, he read still another paper before the New York County Medical Society, on "The Proper Treatment for Laceration of the Cervix," which was subsequently published.§ A few additional papers by Wing,|| Baker,¶ Breisky,** Dudley†† (two), Goodell ‡‡ and Mundé, §§ constituted about allthe published writings upon the subject until the appearance of the recent work of Dr. Emmet (op. cit.), in which it is treated systematically and with great fullness.

Dr Emmet gives a graphic description of the manner in which he detected the presence of the laceration, and the first case upon which he operated. The patient had been an invalid for several years, and had been treated for menorrhagia and hypertrophy of the uterus with an extensive erosion. The latter had, with great care, been healed several times, but a relapse always occurred. Attributing his want of success to the condition of his patient's general health, which was very much impaired, he had almost despaired of affording her any permanent relief. He says:

While making a digital examination one day, I was puzzled to account for the greater width of the cervix in comparison to that of the body

* Principles and Practice of Gynæcology, p. 445.

† *Amer. Jour. Obstetrics*, Feb., 1869.

‡ *Amer. Jour. Obstetrics*, Nov., 1874.

§ *American Practitioner*, Indianapolis, Ind., Jan., 1877.

|| *Bost. Med. and Surg. Jour.*, March, 1876.

¶ *Ibid*, Sept. 27, 1877.

** *Wiener Med. Wochenschrift*, 1876.

†† *New York Med Jour.*, Jan., 1878. *Chicago Med. Jour. and Exam.*, Mar., 1878.

‡‡ "Laceration of the Cervix Uteri." The address in Obstetrics delivered before the Medical Society of the State of Pennsylvania, by Wm. Goodell, A.M., M.D., May, 1879.

§§ *Am. Jour. Obstetrics*, etc., Jan., 1879.

beyond, a condition I had for the first time appreciated. I placed her on the left side, and with Sim's speculum brought the cervix into view. I drew the posterior lip forward toward me with a tenaculum, but with no special purpose, when I was surprised to observe that it had decreased to nearly half its previous size. On lifting up the anterior lip with a tenaculum in the other hand, so as to bring the two portions in approximation, the outline of a cervix presented, of nearly a normal size. The difficulty was at once apparent, for the parts had rolled back within the uterine canal, and a deep lateral fissure became evident, which extended on each side entirely through the cervix and beyond the vaginal junction. On separating the flaps and forcing them back to their former position, I saw the tissues gradually roll out and the cervix again present its previous appearance. There could then be detected no appearance of laceration, and with the reduplication of the vaginal tissue over the sides of the uterus, as I have already described, the cervix presented a normal length above its apparent junction with the vagina. The remedy at once suggested itself; the operation was performed. * * * On completing the operation, the uterus was five inches in depth; it rapidly reduced in size, and in time all evidence of local disease subsided.

My own attention was called to this subject more than four years ago, in connection with a case which was almost the counterpart of the one just related.

Mrs M., thirty-two years of age, the mother of three children, the youngest of whom was five years old, had been under my care about two years, with occasional intermissions. She had become as tired of me as I was of her, when one day she asked me whether I thought she could be cured if she went to New York. I felt piqued by the question, which aroused in me a fresh interest in her condition. I had a short time before been reading Dr. Emmet's paper, already referred to, on the subject of laceration of the cervix, and it occurred to me to ascertain whether my patient might possibly be suffering from such an injury. Accordingly I placed her in the knee-chest position, and introduced a perineal elevator into the vagina. The latter became widely distended, and I was afforded a full view of its anterior and lateral walls, and the vaginal portion of the cervix. But the parts appeared so differently from what I had been accustomed to see, that I almost doubted their identity. Prior to this time I had always examined her in the dorsal position, the head and shoulders being raised. In that posture, and also when the patient was on her feet, the lower part of the uterus was found just within the vulva, and a finger could be passed into the vagina to the depth of two and a half inches before it reached the apparent utero-vaginal junction. Now, however, as the weight of the uterus carried it from the vulva and drew out the vaginal walls to their full length, I saw that the erosion, instead of appearing, as formerly, to involve a flattish surface nearly two inches in diameter at the end of an elongated cervix, was depressed in the middle, and reached not only to but beyond the vaginal junction; while the vaginal portion of the cervix was actually but little longer than it should be. Subsequently, aided by an assistant, I brought the anterior and posterior flaps together with tenacula, and the red eroded tissues were rolled in out of sight, and the mushroom shaped thickening of the cervix at once disappeared. In short, it was evident that I had before me a typical case of bilateral laceration of the cervix uteri, which I had been typically maltreating for something else. I had been trying, *secundum artem*, to cure this patient of a granular erosion of the os uteri, with enlargement and elongation of the cervix uteri, prop erly speaking, neither of these conditions were present, or at least, only as symptoms.

Although this was the first case in which I discovered the lesion, it was not the first in which I operated; for, before I could obtain the consent of Mrs. M.—or rather that of her friends—to have an operation performed, I had succesfully operated upon another lady in whom, guided by my experience in the former case, I diagnosticated a laceration at the first examination.

Down to the present time I have operated twenty-three times for this malady. In one case the operation was followed by a rather severe attack of pelvic cellulitis, from which, however, the patient recovered, and the operation succeeded. In another case, there was secondary hæmorrhage, requiring the vaginal tampon. In three cases the parts failed to unite—in two completely, in one partly, results due, I believe, to the lack of sufficient and proper preparatory treatment. In two of these, a second operation was successful; the third declined further treatment.

*Frequency.*—The experience of Dr. Emmet, based upon 500 cases, observed in private practice leads him to affirm that 32.80 per cent., that is, nearly one-third, of "all women who had been impregnated, and had suffered from some form of uterine disease, were found to have laceration of the cervix;" and he concludes his consideration of the subject by stating that "at least one-half of the ailments among those who have borne children are to be attributed to laceration of the cervix."*

While I believe that there are few who would go so far as to endorse this statement unconditionally, I am of the opinion that, if all cases of laceration, slight and severe, be included, he

* Loc. Cit., p. 480.

is not wide of the mark; certainly, he is more nearly correct than are those who regard the injury as of rare occurrence. The conclusions of other observers whose attention has been directed to this point, do not greatly differ from those of Emmet. Thus, Dr. P. F. Mundé* found 119 lacerations in 700 women examined; and Dr. Goodell † says, "My own experience at the Dispensary for Diseases of Women at the University of Pennsylvania, would lead me to infer that about one out of every six women suffering from uterine trouble has an ununited laceration of the cervix."

I am aware that this great frequency of the lesion has been denied, but it is evident from what has already been advanced, that any one who has not given special attention to it—who has not looked for it—and who, above all, has not adopted the proper means for detecting it, can hardly be in a position to form a just opinion upon the question.

*Causes.*—Previous organic disease of the cervical uterine tissues doubtless predisposes to the accident. Cervical metritis, for example, whether in its soft or indurated stage would interfere during parturition more or less efficiently with the gradual and uniform dilatation of the parts upon which the integrity of these latter depend. So, likewise, would the rigid cicatricial condition of the os and cervix, which is so frequently found as the result of the long continued application of nitrate of silver, caustics, etc., for real or fancied disease.

Among the exciting causes may be ranked obstetric operations, as turning, the use of forceps, etc., not necessarily from carelessness or lack of skill on the part of the obstetrician, but because these manœuvres require a certain amount of manual force, and also because of the state of the parts which frequently makes their employment necessary. Of course, such an untoward result is more likely to follow these procedures if they be attempted before the os uteri is in a suitable condition of dilatation or dilatability. A laceration may likewise occur in consequence of an undue violence in delivering the placenta. Other causes of this character are, the administration of ergot in unsuitable cases, especially before dilatation has been accomplished; the premature rupture of the fœtal membranes, whereby their soft and insinuating pressure is replaced by the more unyielding head.

I have had the impression that rapid labors, by not affording sufficient time for normal dilatation, would be found to be a cause of laceration, but an examination of the tables furnished by Dr. Emmet, seems to show that such is not the case. In 164 cases in which the character of the labor was ascertained, it was rapid in 30, tedious in 50 and natural in 21.

*Varieties.*—The injury varies both in direction and extent. It may involve only the anterior or posterior lip or both; it may be transverse or oblique. The rent is not always in a straight line, but is sometimes curvilinear, or at least it assumes this direction during the healing process.

In extent, too, it varies greatly; in some cases involving only the edge of the os uteri to the depth of a quarter or third of an inch, but in others passing quite across the crown of the cervix, and in still others extending to and beyond the vaginal junction. Dr. Emmet * mentions several instances in which the rent, passing the cervical limits invaded the bladder and posterior cul-de-sac respectively, leaving as a consequence, in the former case, either an extensive fistula involving all the injured parts, or, where partial spontaneous union subsequently occurred, either a small vesico-vaginal fistula just in front of the cervix, or a vesico-utero fistula, through which urine might escape from the bladder into the uterine canal at a point as high as the internal os uteri or even above.

Lacerations which do not extend to the vaginal junction are called incomplete, while those which reach to or above that point are said to be complete.

There is another variety of the injury besides those mentioned which may also be termed incomplete. It is that in which the rent does not involve the entire thickness of the cervical wall. Emmet describes these cases as follows: "It would seem as if partial laceration took place from the internal os downward on different sides through the mucous membrane and deeper tissues, without extending to the vaginal surface of the cervix, making

* Vesico-Vaginal Fistula, etc. By Thos. Addis Emmet, M.D. Wm. Wood & Co., New York.

folds not unlike those between the ribs of a partially opened umbrella, which disappear when it is fully opened out. Through the patulous os and canal the mucous membrane is seen prolapsed, and its appearance is like that presented after dilating with a sponge tent a partial contraction of the canal which had taken place above, but had not yet extended to the external os. The cervix is frequently but little enlarged in diameter, but its walls are seen to be thinner than natural."

Yet other varieties than those enumerated result from the number of fissures. If there be but one, to the right or left, it is said to be unilateral; while, if there be one on each side it is bilateral; should there be still more, trilateral, stellate, etc.

Of all the varieties of laceration, Dr. Emmet believes that those in the antero-posterior direction are the most frequent, but that, owing to the fact that the patient is enforced to maintain the recumbent position for some time after labor, the pressure of the lateral walls of the vagina maintains the torn edges in contact until union occurs, leaving only a cicatricial line to mark the site of the injury. My own experience, much more limited, certainly, does not, however, coincide with that opinion. Although since my attention was first called to this subject, I have carefully looked for the evidences of previous laceration, I have not found them nearly so often in the anterior and posterior lips as in a transverse direction; and I think we might in advance suppose that this should be the case from the fact that the cervical walls are thinner in this direction than in the other. However this may be, it is quite certain that lacerations through the anterior or posterior lip are much more apt to heal spontaneously than are those in other directions, because of the lateral pressure of the vaginal walls; and, also, that even where spontaneous union does not take place, the same force tends to present the serious consequences which result from the eversion of the cervical lining which so much more commonly occurs in the other varieties of cervical laceration, especially the bilateral, provided they be of sufficient extent.

*Progress, Symptoms, etc.*—Lacerations which involve only the edges of the os uteri frequently, perhaps generally, heal spontaneously before the woman leaves her bed.

Those which occur in a forward or backward direction, even when complete, may likewise unite and leave little or no trace, the raw surfaces being kept in contact in the manner already indicated.

Most of the cases met with in practice are those in which the laceration is in a transverse direction, and in which it has passed beyond the crown of the cervix. In nearly all of these, especially where the rent has extended to or beyond the vaginal juncture — the intra-cervical membrane becomes everted. Its delicate covering accustomed to contact with only its own alkaline secretion is now constantly bathed with the acid secretion of the vagina, which acting as an irritant, soon causes removal of the epithelium. This abnormal state of the parts prevents or retards the proper involution of the uterus, which, consequently, remains enlarged and soft. All these unfavorable conditions are increased so soon as the woman quits the bed and gets upon her feet. Then the heavy uterus, inadequately sustained by its supports, presses upon the floor of the pelvis. The intra-cervical surface of the posterior flap of the laceration impinges upon the corresponding vaginal wall, while the anterior flap is pushed toward the vulva. The everted and eroded surface now suffers additional irritation from the pressure and chafing to which it is subjected. Under these circumstances the flaps are forced farther and farther apart; the extruded lining membrane, by reason of its interrupted circulation, becomes thickened and congested, and thus assists in its own out-rolling. The uterus being in a state of fatty degeneration (the first stage of involution only having been accomplished), the flaps are soft and yielding. Their inner surfaces are hence readily flattened out against the vaginal walls, and the entire cervix finally is everted, the internal os uteri becoming the lowest portion of the organ, and appearing at what seems, owing to the reflection of the vaginal tissue, a very long and very broad crevix.

The nabothian glands, thus exposed, are at first stimulated to undue activity, as evinced by the greatly increased quantity of secretion poured out by them, and which constitutes the glairy, tenacious discharge, which is so characteristic of endo-cervical inflammation. By and by, they become diseased. Their excretory ducts close, and their contents being thus retained, they enlarge and form protruding roundish masses, varying in size from that of a millet-seed to that of a grain of barley. Some of these rupture, and their inclosed mouths present an appearance of increased roughness and rawness to the already eroded surface. Before bursting they may frequently be felt as small shot-like bodies in the tissues.

When the injury is confined to one side, the eversion of the cervical lining is not so marked, and occasionally does not occur at all, even when the rent has extended to the vaginal junction. In these cases there has usually taken place some degree of union at the bottom of the fissure, and the cicatrix thus formed restrains, partially or wholly, the tendency to protrusion.

*Symptoms.*—The symptoms produced by laceration of the cervix consist of various pains in the back, hips and thighs; a sense of weight and "dragging" in the pelvis increased in severity when the patient is in the standing posture, or has undergone any unusual amount of fatigue. Leucorrhœa is nearly always present, and the discharge is sometimes tinged with blood. Menstruation is frequently though not always disturbed. Usually the flow is increased in quantity, and appears after shortened intervals; metrorrhagia is also not infrequent. On the contrary, in a smaller number of cases the menstrual discharge is diminished in quantity. Sexual appetite is frequently impaired, and sometimes abolished entirely. Neuralgic pains referred to the region of the cervix, sometimes of great intensity, are present in some instances.

In the worst class of cases no long time elapses before the lack of exercise and the persistence of pain and exhausting discharges, produce their legitimate results upon the general health. Digestion is impaired, the appetite fails, the bowels become constipated, assimilation is interfered with. A lessened supply of impoverished blood produces pallor and sallowness of complexion, debility, disturbed and insufficient sleep. The stomach, liver, bowels, kidneys and especially the bladder and rectum all contribute their quota of sympathetic manifestions and combine to render the woman's life utterly wretched.

One of the notable effects of the injury as pointed out by Dr.

Goodell * is that of sterility. He says, "This lesion is so common a cause of sterility that I always suspect its existence whenever a guileless woman stops bearing after her first labor."

As a rule, the severity of the symptoms, local and general, resulting from laceration of the cervix, are in direct proportion to the extent of the injury, and especially to the amount of ectropium present. But this is by no means always the case. I have known some instances in which the laceration was of very moderate extent, and in which only a small portion of the cervical lining membrane was extruded, and yet the patients were obliged to be in bed the greater part of the time on account of uterine and ovarian neuralgia, dysmenorrhœa, pains in the back and pelvis, general anæmia, etc.; while, on the other hand, I have in mind one case in which, with a transverse laceration extending on both sides from the os uteri quite to the vaginal junction, with accompanying extensive eversion, the woman was able to perform her usual household duties. These anomalies of the relation between cause and effect are not, however, as is well known, confined to the injury under consideration. The history of disease —of pelvic disease, particularly—is full of them. The extent to which women suffer from a given amount of disease or injury of the reproductive organs is greatly influenced by the social condition and surroundings of the patient. Those in the higher ranks of life, who live luxuriously, whose nervous systems have been unduly and unsystematically developed at the expense of the osseous and muscular, suffer most, for in them is engendered an unnatural tendency to irritability and emotional and sensational disorders.

*Diagnosis.*—It would seem that, from the accessibility of the parts both to touch and sight, nothing could be easier than the recognition of a laceration of the cervix uteri; but the fact that it eluded discovery by so many expert and earnest workers for so many years, shows that what appears to be so simple a matter is really not so. And, if any one should still entertain a doubt upon this point, Dr. Emmet's testimony is surely sufficient to remove it. He says,† "It was fully six years after my first

* Loc. Cit.

† Principles and Practice of Gynæcology, p. 447.

operation before I had gained experience enough to detect this lesion under its varied forms."

No doubt the tardy recognition of the injury, and the difficulties attending its diagnosis may be explained by the fact that ocular examination of the parts is usually made with tubular or valvular specula; and these instruments, especially the former, by their peculiar action prevent rather than facilitate the revelation of the condition. Another circumstance which leads to error is that women are commonly examined while lying on the back, with the head and shoulders elevated. In this position the heavy uterus is found low down in the pelvis, and, carrying with it a reflection of the vagina, the vaginal portion of the cervix acquires a fictitious elongation, to the extent, sometimes, of more that two inches. At the same time the forces which separate the flaps of the laceration also cause these latter to present a flattened surface of eroded tissue from one to two inches across, with the apparent os uteri externum in the center. Looked at in this state, all appearance of laceration is lost.

The conditions with which laceration is liable to be confounded are, simple granular erosion, thickening and elongation of the cervix, ulceration and epitheloma.

Erosion of the os and cervix uncomplicated with laceration, is usually caused by the irritating contact of uterine catarrhal discharges; and to show how comparatively rare this condition is, I may mention that, among the 700 women examined by Mundé, and who furnished 119 cases of erosion *with* laceration, there were but (11) eleven cases *without* that complication.

Where there is actual enlargement of the cervix, the part is gradually increased in diameter from below upwards towards the body of the organ; whereas, when a laceration with eversion is present the lowest portion is the largest; and between it and the part above there is formed a more or less distinct neck or narrowed portion, so that its shape has been compared to that of an inverted mushroom. Hence, when by the touch this constricted portion can be felt, a laceration may be suspected.

I do not wish to be understood as implying that there may not be actual enlargement of the cervix when the latter has been lacerated. In point of fact, the irritation to which, in cases of marked eversion, the cervical lining is subjected, the cystic degeneration of its glands, and the continually increasing obstruction to its circulation combine to produce what Klob denominates a "formative irritation," which results in increased growth.

But the touch is not sufficient to enable us to distinguish a laceration from true elongation of the cervix, a condition, by the way, which Emmet affirms has no existence. Whether in this he be correct or not, the *apparent* elongation of the cervix which is present in some cases of laceration may always be made to disappear by placing the patient in the knee-chest position. The superincumbent pressure of the intestines being thus removed, and the uterus permitted to fall *away* from the pelvic floor rather than *toward* it, the vagina is pulled out to its full length, including the reflected portion which has been carried down by the cervix, and the true cervico-vaginal junction is at once seen.

Any one who has observed the appearance presented by the red, roughened, eroded cervical lining in a case of laceration of the cervix, need not be told how easy it is to mistake it for a true ulceration of the part. But, if by the term ulceration of the cervix we mean to imply a loss of substance other than epithelial, it is a comparatively rare condition. The only true ulcers of this part are those produced by friction when the uterus is procident, or which are the result of chancroid or carcino-matous disease.

The extruded lining membrane studded with enlarged follicles, elevated and swollen, bleeding readily, and bathed with muco-purulent secretion has not infrequently been mistaken for malignant disease, and, it is reasonable to suppose, that some of the cases of reported permanent cure by amputation, cautery, etc., of supposed carcinomatous enlargement of the cervix, have really been cases of laceration with extensive eversions.

Now, from all other conditions presenting a red, eroded or ulcerated surface, and from all those which are accompanied by enlargement, laceration may be distinguished with the utmost certainty and precision. It is done as follows: Place the patient in the semi-prone position, or, still better, the knee-chest position. Introduce Sim's duck-bill speculum or other form of perineal retractor, and when the vagina is sufficiently distended insert small tenacula into the sound mucous membrane in front and behind, just beyond the edge of the eroded surface. If now, by approximating the points of the tenacula the erosion can be rolled into the cervical canal, and the vaginal portion be made at the same time to present a comparatively normal shape and size, it is a laceration. With no other condition can this manœuvre be made with these results.

If the laceration has healed without union, and there be left a fissure to mark its site, there can be no difficulty in recognizing it. But this result does not commonly occur unless the laceration has been unilateral; and these cases, so easily detected, are not of great pathological importance, for they are not attended by the out-rolling of the cervical lining which is generally the essential cause of the symptoms resulting from the injury. In by far the greater number of bad cases no cleft or fissure whatever is apparent either to sight or touch.

*Treatment.*—Many of the distressing symptoms, backache, pelvic pains, leucorrhœa, may be greatly benefited by rest in the recumbent position, vaginal injections of hot water, glycerine tampons, astringent and caustic applications, etc. By persistence in the use of these means in conjunction with the internal administration of ergot, strychnia, iron, etc., and such other constitutional remedies as may be indicated, patients afflicted with laceration, even in some of its severer forms, may be kept in a fairly comfortable condition; and where the injury is slight and unaccompanied with marked eversion, a permanent cure may sometimes be effected. Usually, however, soon after their use is discontinued, and the patient resumes her ordinary duties, the former symptoms manifest themselves anew, and she becomes as miserable as before. This result is quite certain to occur if the injury has been so extensive as to permit much eversion.

When, in consequence of its increased size and weight the uterus has become retroverted and prolapsed, much relief may sometimes be obtained by raising the organ to its proper position and supporting it with a suitable pessary. This not only serves to lessen the uterine congestion by promoting the emptying of the uterine veins and sinuses, but it also prevents the extruded and eroded mucous membrane from pressing and rubbing against the vaginal walls. However, the use of pessaries is not always feasible. In some cases there is present so great a degree of tenderness in and about the uterus as to preclude the slightest pressure from an instrument, while in others the cul-de-sac is so effaced by the contraction of cicatricial bands and post-uterine inflammatory deposits as to leave no resting place for the upper end of the pessary, which, consequently, has a constant tendency to slip forward over the cervix and take a position between it and the bladder. In this position it not only ceases to be of benefit but is positively hurtful.

I may say, in passing, that I regard those cases of retroflexion and retroversion of the womb as practically incurable in which the body or fundus is bound down by adhesions, or where, without adhesions, the displacement is accompanied either by a very shallow cul-de-sac or a very short vaginal portion.

When the use of a pessary is indicated I prefer a Hodge closed lever, or some one of its modifications, especially that of Dr. Albert Smith.

For the relief of the tender and painful condition of the parts just referred to, I have found the following plan satisfactory: Once a week I abstract from the cervix a half ounce to two ounces of blood, by numerous punctures with Buttle's lance. The punctures are made directly into the eroded mucous tissue and into any cystic glands that may be apparent. When these latter are small and very abundant short incisions are preferable to punctures, as they are less likely to close subsequently. The operation is followed by the application to the cervix of a glycerine tampon on which has been sprinkled two or three grains of iodoform. When the odor of this agent is objected to I replace it with one grain of sulphate of morphine. In addition, I direct a hot water vaginal injection to be used every night at bedtime—except on the day of scarification—and a glycerine tampon unmedicated, to be placed every morning. All this local treatment is suspended during the menstrual period. I need hardly say that the blood-letting, even to the slight extent advised, is contra-indicated in the case of patients who are anæmic and in whom the blood-making power is impaired.

By the judicious use of these means for a few weeks we may frequently be enabled to use a small pessary (the instrument should never be large) where at first it would have been impossible to do so.

But, as already intimated, all the foregoing means are at best uncertain, always tedious, and generally ineffectual. It is fortunate, therefore, that we have a remedy which is both safe and certain; one which not only removes the symptoms of the disorder but which restores the parts to their normal condition.

I refer to the plastic operation devised by Emmet, and to which my friend Dr. E. C. Dudley has given the distinctive name *trachelorrhaphy*.

This operation is one of the most successful with which I am acquainted, and when its simplicity and freedom from danger are considered in relation to the amount of benefit which it is capable of conferring, one of the most useful in uterine surgery.

*Treatment preparatory to the Operation.*—In most cases where the operation is decided upon, some amount of preparatory treatment is beneficial and in some it is absolutely necessary.

It should be an inflexible rule in plastic surgery to have the parts in a healthy condition, if possible, before any operation be attempted, otherwise full success cannot reasonably be expected. To lack of attention to this rule I think may be fairly attributed many of the failures of operations undertaken for the cure of vesico-vaginal fistula, ruptured perineum, vesicocele, etc. In lacerations of the cervix it is not always possible to bring the parts into this desirable state of local health, because their displacement is incompatible with it; still it may always be approximated.

The proper preparatory measures should be such as tend to remove or lessen uterine congestion; as the daily hot vaginal douche, the evacuation of the cervical cysts, the local obstraction of blood, the glycerine tampon, etc.; in short, just such means as have been detailed under the head of palliative treatment. No attempt should be made to glaze over the eroded surface with the solid nitrate of silver, formerly, and, I fear still, the common practice. The improvement produced in the appearance of the part by this measure is fallacious. The dense cicatrical tissue thus formed, by enclosing and compressing the peripheral nerve filaments almost invariably causes uterine and ovarian neuralgias, diminishes or destroys sexual appetite, and leads to various other nervous and psychological disturbances.—(Goodell).

The existence of marked tenderness as a result of inflammation in the neighborhood of the uterus or broad ligaments, is a contra-indication to an operation ; but the presence of old inflammatory exudations which are not the seat of tenderness or pain, I do not consider so, provided there be no present uterine congestion. For the operation, if carefully performed, without dragging the uterus from its position, is less likely to re-establish fresh inflammation than is the continuance of the laceration. I have operated two or three times under these circumstances without any untoward result.

The best time for the operation is between the third and seventh day after a menstrual period; as the pelvic organs are then physiologically more quiescent than at any other time, and besides, sufficient time is afforded them to recover entirely before the onset of the next period.

The patient should take a laxative dose of medicine in the afternoon of the day preceding that of the operation; and I find it well also to have an enema of warm water given on the following morning — say an hour before the operation.

*Operation.* — All being in readiness, the operation may be performed as follows : The patient, being etherized, is placed on her back on a firm table, which need not be more than four feet long. She is drawn forward so that the hips project three or four inches beyond the edge, and each lower limb is given in charge of an assistant, by whom the thighs are flexed upon the abdomen. The operator then passes one or two fingers of the left hand into the vagina, and with the right introduces the blade of a perineal retractor. (I prefer one of this sort for the purpose : it is a modification of that of Simon, and has a much shorter and much flatter blade than Sim's duck-bill.) This is guided to its position in the posterior cul-de-sac by the fingers in the vagina, and the perineum being pressed downwards while the vaginal walls are separated by the fingers, the cervix is readily brought into view.

One of the assistants now takes charge of the retractor with his disengaged hand. The anterior flap of the laceration is seized with a small double-hooked vulsellum, and, aided if necessary by supra-pubic pressure, the lower portion of the uterus is brought quite to the vulva. Both flaps are now approximated, with the view of determining accurately the proper position of the external os uteri, the extent of the laceration, the quantity of tissue to be removed, the number of stitches requisite, etc. These points having been settled, a curved or rectangular needle, fixed in a handle, and having its eye near the point, is passed through the posterior flap at a point which is to mark the middle of the os uteri; it carries a strong silk suture, the loop of which is seized when it appears, and the suture is left in the flap on the withdrawal of the needle. The same process is repeated with the anterior flap. By means of these sutures, complete control of the cervix is had throughout all the subsequent steps of the operation. They should be sufficiently long to enable the assistant who has them in charge to draw them in any needed direction, without at all incommoding the operator.* I formerly used a single thread, which passed through both lips, but this prevented the separation of the latter when they were drawn upon, and I find it much more convenient to transfix each separately, so that either lip can be acted upon independently of the other.

The surfaces of the parts to be joined together are next made raw. I prefer doing this with scissors, as being a more expeditious and less bloody mode than that by the knife. The thickness of tissue to be removed depends somewhat upon the shape of the flaps. If their inner surface be full and rounded, the convexity should be wholly ablated. A safe rule is to remove enough to permit the opposing sides to be brought *easily* into contact, so that there may be no undue strain upon the stitches. Emmet lays much stress upon the necessity of removing all cicatricial tissue, regarding its presence as a frequent cause of neuralgia. Care should be taken to not freshen the surfaces too near the central portion of the flaps, lest their subsequent adhesion should result in obliteration or narrowing of the external os. A space

* I find that Dr. Goodell makes use of a similar device. He "passes on each side of it" (the os externum), "through both lips of the cervix, a long iron-wire suture."

at least one-half inch long should be left for this opening; for the subsequent shrinking of the part will reduce this to about the proper size. Indeed, a still more liberal provision for the outlet ought to be made, if there be present very great hypertrophy of the uterus.

The opposing denuded surfaces should be equal in length and width, so that when brought together coaptation may be accurately made.

Usually but little hæmorrhage attends the freshening of the tissues, although in some cases it is sufficiently profuse to delay the operation somewhat. The only artery likely to be injured is the circular, which passes near the vaginal junction, sometimes just within the outer angle of the fissure, and great care should therefore be taken to not cut deeply when paring the edges at this point. The capillary bleeding commonly ceases directly the sutures are tightened.

The passing of the sutures is sometimes very difficult, owing to the thickness and density of the tissues. It is necessary to have a strong needle holder, one which permits the needle to be placed at any angle. The needles should have a cutting edge near the point, and should vary in length from three-fourths of an inch to an inch and a quarter; they should be thick enough to allow of a large eye through which the loop of a doubled silk thread may pass. The eyes of most needles are too small.

The first suture is passed at or near the inner angle of the fissure. The part being steadied by means of a firm tissue-holder, the needle is entered about a quarter of an inch from the edge of the freshened surface, and made to emerge just at the edge of the undenuded strip which has been left to form the continuation of the uterine canal; it is then re-entered at the corresponding point opposite, and, passing through the other flap, finally issues at a point which corresponds with that of its first entrance. A silver wire of suitable length is then connected with the loop, and is left in place on withdrawal of the needle, in the usual manner. It is immaterial through which flap the needle is passed first. The number of sutures required will vary according to the length of the fissure. Usually, from two to four are needed on each side. They should be placed about one-third of an inch apart.

In my more recent operations I have adopted a different plan from that just described, for passing the sutures, in all cases in which the cervix could be brought sufficiently within reach ; that is, I have used a small perineal needle in a fixed handle, with the eye near the point. With this I transfix both flaps, and when the eye of the needle emerges from the second one, it is directly threaded with the silver suture. The needle is then withdrawn, leaving the wire in place. In this way the sutures are inserted with great accuracy and rapidity.

Either silk or silver wire may be used for the sutures. I prefer the wire, because, perhaps, I am accustomed to it, and know it to be reliable. Dr. A. J. Skene,* of Brooklyn, reports that he successfully uses silk, in every case obtaining a satisfactory result. If, upon further trial, silk be found equally trustworthy with the wire, the operation can be more quickly and easily done.

The sutures having all been inserted, the patient should be placed in bed, where, ordinarily, she ought to remain until the stitches are removed. This may be done as early as the eighth day, but I prefer, unless the prolonged confinement be especially irksome, or contra-indicated, to allow them to remain until the ninth or tenth. In one case, in which they were removed on the eighth day, the adhesion of the flaps was so feeble that they subsequently separated, and a repetition of the operation became necessary.

The operation is not a very painful one, and an anæsthetic, although desirable, is not always necessary. I operated on one patient without using any, and she subsequently stated that she suffered less from the pain of the procedures than she had on a former occasion from the nausea and vomiting following the inhalation of chloroform for the extraction of a tooth. And, in another case, in which Dr. Dudley kindly operated at my request, to exemplify the manner of Emmet, no anæsthetic was given, and although the operation lasted a full hour, the patient only com-

* Proceedings of the Medical Society of the County of Kings, June, 1878.

plained of the constrained position and the action of the Sim speculum.

Neither is there much pain or discomfort felt after the operation. Indeed, patients usually suffer so little from it that it is difficult to make them appreciate the necessity for lying in bed afterwards. And it is possible that this necessity has been overrated. Several times patients have, contrary to my directions, resumed many of their ordinary duties after the third day; in one case, on the second morning after the operation, I found the patient dressed and sitting up, and she informed me that she felt less discomfort in the parts than she habitually did before the operation. In none of these cases did the seeming imprudence produce any bad result or prevent a successful issue. Dr. Skene states that he has operated eight times at his office and sent the patients home in the street cars.

*After Treatment.*—At the close of the operation I am in the habit of placing in the vagina the ordinary cotton tampon saturated with glycerine, with a string attached to facilitate removal; and in the rectum a suppository of cocoa-butter, containing two grains of opium and one-sixth of a grain of extract belladonna. The tampon should be removed on the day following the operation, and the vagina should be carefully washed once or twice daily with warm water slightly carbolized. It is not necessary to prohibit the patient from leaving the bed to urinate, or for other necessary purpose. Rarely I have had to use the catheter once or twice. The bowels, if not open spontaneously, may be acted upon by a mild laxative, followed by an enema on the fourth or fifth day.

Among those who have investigated this subject and who have learned to recognize and treat the lesion — with few exceptions — there is no longer any doubt as to the safety, the efficiency, and in cases of *complete* laceration, of the necessity of hystero-trachelorraphy, as a means of cure. But there are a vast number of cases in which the laceration has been slight, in which the rent has not extended beyond the vaginal junction, perhaps only involving the crown of the cervix. In many of these cases the mucosa is only slightly everted, and there is no such apparent elongation or enlargement of the cervix as is seen in the more typical cases. But the patient suffers from leucorrhœa, backache, possibly menorrhagia, and may present marked nervous disturbance. What should be done for these cases? These are the milder forms of "ulceration," and it is the experience of all gynæcologists that they may be cured by topical applications of an astringent and caustic nature. But the treatment by these means is tedious, usually covering several months and sometimes is not successful. Iodine, carbolic acid, nitrate of silver, iron and similar applications fail very often to do anything more than temporarily ameliorate the patient's condition. After each menstrual period she is usually found to have relapsed more or less, and after exhausting her patience—perhaps her purse, also—and the ingenuity of one doctor after another, she finally ceases her efforts and accepts a life of invalidism as best she may. Even in the most successful of these cases, several weeks of uninterrupted treatment are necessary to the cure. Cannot and should not these cases, like the more severe forms of laceration, be treated by the operation?

Dr. P .F. Mundé, in an admirable paper, already alluded to * advocates the practice on the ground that by means of the operation such cases can be more quickly and more certainly cured than by the methods in common use, and he adds by way of final argument: "Another reason why trifling ectropia should not be allowed to go on for years unheeded and untreated, was recently advanced by Prof. Breisky,† of Prague, who has observed and operated upon four cases of laceration, in which one of the everted lips had become carcinomatous in consequence of the irritation to which the exposed cervical mucosa was subjected." These observations are confirmed by Veit,‡ who, out of nine cases of carcinomatous cervices found three in which the disease originated in the enlarged glandular elements.

At a meeting of the German Gynæcological Society, held at Cassel, Sep. 12th and 13th, 1878, § Kugelman, of Hanover, expressed his disapproval of operative measures for the cure of such

* Indications for Hystero-Trachelorrhaphy. Amer. Jour. Obs., June, 1879.

† Wiener Med-Chirurg. Rundschau, Aug. 1877.

‡ Gyn. Sec. German Congress of Phys., 1877.

§ See Amer. Jour. Obs., Jan., 1879.

uterine ailments as were amenable to other less severe means of treatment, although the latter might require "much time and patience." In reply to this, Prof. Schröder said that "the patient, as well as the physician, needed time and patience," and that it was surely better for her to be freed from the trouble in a fortnight, by a safe and certain method, than to pass through months of treatment for an uncertain result.

